# Modulation of urelumab glycosylation separates immune stimulatory activity from organ toxicity

**DOI:** 10.3389/fimmu.2022.970290

**Published:** 2022-09-29

**Authors:** Carmen Reitinger, Andrea Ipsen-Escobedo, Chiara Hornung, Lukas Heger, Diana Dudziak, Anja Lux, Falk Nimmerjahn

**Affiliations:** ^1^ Chair of Genetics, Department of Biology, Friedrich Alexander University of Erlangen-Nürnberg, Erlangen, Germany; ^2^ Laboratory of Dendritic Cell Biology, Department of Dermatology, University Hospital Erlangen, Erlangen, Germany; ^3^ Medical Immunology Campus Erlangen, Erlangen, Germany; ^4^ Deutsches Zentrum Immuntherapie (DZI), Erlangen, Germany; ^5^ Comprehensive Cancer Center Erlangen-European Metropolitan Area of Nuremberg (CCC ER-EMN), Erlangen, Germany

**Keywords:** CD137, Fc-receptors, glycosylation, therapeutic antibody, urelumab

## Abstract

Checkpoint control and immunomodulatory antibodies have become important tools for modulating tumor or self-reactive immune responses. A major issue preventing to make full use of the potential of these immunomodulatory antibodies are the severe side-effects, ranging from systemic cytokine release syndrome to organ-specific toxicities. The IgG Fc-portion has been demonstrated to contribute to both, the desired as well as the undesired antibody activities of checkpoint control and immunomodulatory antibodies *via* binding to cellular Fcγ-receptors (FcγR). Thus, choosing IgG subclasses, such as human IgG4, with a low ability to interact with FcγRs has been identified as a potential strategy to limit FcγR or complement pathway dependent side-effects. However, even immunomodulatory antibodies on the human IgG4 background may interact with cellular FcγRs and show dose limiting toxicities. By using a humanized mouse model allowing to study the immunomodulatory activity of human checkpoint control antibodies *in vivo*, we demonstrate that deglycosylation of the CD137-specific IgG4 antibody urelumab results in an amelioration of liver toxicity, while maintaining T cell stimulatory activity. In addition, our results emphasize that antibody dosing impacts the separation of side-effects of urelumab from its therapeutic activity *via* IgG deglycosylation. Thus, glycoengineering of human IgG4 antibodies may be a possible approach to limit collateral damage by immunomodulatory antibodies and allow for a greater therapeutic window of opportunity.

## Introduction

Monoclonal antibodies have become crucial therapeutic agents for the treatment of human cancer and autoimmune diseases and novel monoclonal antibodies are being developed continuously (1). In addition to cytotoxic antibodies, such as rituximab or herceptin, which recognize antigens expressed on tumor cells, antibodies aiming at harnessing tumor specific T cell responses have revolutionized the field of antibody-based cancer immunotherapy (2, 3). This class of antibodies is referred to as checkpoint blockade or immunomodulatory antibodies and contains antibodies specific for CTLA-4, PD-1, PD-L1, CD137, Ox40, GITR, or CD40 expressed on T cells or antigen presenting cells. Of note, some immunomodulatory antibodies directed against CTLA-4 or CD137 also show promise for modulating self-directed immune responses in pre-clinical model systems but also in patients with autoimmune diseases, broadening the therapeutic value of this class of molecules beyond the treatment of cancer (4–6).

A major factor restricting the therapeutic window of checkpoint control or immunomodulatory antibodies are the severe side-effects triggered upon antibody infusion, ranging from an acute cytokine storm to organ specific autoimmunity affecting the gut and liver, for example (7, 8). To circumvent systemic side-effects, an intra-tumoral injection of immunomodulatory antibodies may be a rescue strategy limited, however, to accessible tumor entities (9). To understand the activity of immunomodulatory antibodies in more detail, several groups have studied informative pre-clinical model systems. These studies have emphasized that the Fc-domain of various immunomodulatory antibodies can play a major role for antibody activity *in vivo*. For example, antibodies targeting molecules expressed on regulatory (T_reg_) T cells, including CTLA-4, GITR, OX40, and CD137, have been shown to act as cytotoxic antibodies and deplete T_reg_ cells within the tumor microenvironment *via* binding to activating Fcγ-receptors (FcγRs) (10–14). Further along these lines, an optimal activity of CD40-specific antibodies required the *in vivo* cross-linking of these antibodies *via* the inhibitory FcγRIIb (15, 16). Alternatively, human IgG subclasses, such as IgG2, allowing for an optimal CD40 cross-linking through unique antibody isotype intrinsic features could circumvent the requirement for higher order cross-linking through neighbouring FcγRIIb expressing cells to achieve super-agonistic activity (17, 18). For immunomodulatory antibodies not requiring or not intended to have an IgG Fc-domain dependent enhancement of therapeutic activity, the use of Fc-domains, such as human IgG4, allowing to maintain a long antibody half-life while limiting the interaction with the complement or FcγR system have become the format of choice. This includes antibodies such as pembrolizumab or urelumab, targeting PD-1 or CD137 on T cells, respectively. Indeed, in mice a PD-1 antibody variant carrying an IgG2a Fc-domain allowing an optimal interaction with the FcγR system resulted in a reduced therapeutic activity *in vivo*, due to the elimination of intratumoral cytotoxic T cells (19). Further along these lines, CD137 (4-1BB)-specific antibodies on a mouse IgG2a backbone, efficiently depleted activated T cells and T_reg_ cells *via* activating FcγRs, while mouse IgG1 variants of the same antibody stimulated cytotoxic T cell responses *via* the inhibitory FcγRIIb (5, 10, 20, 21). However, several studies have shown that human IgG4 antibodies may productively interact with human FcγRs *in vitro* and *in vivo*, suggesting that human IgG4 Fc-domains are not inert and may contribute to wanted and unwanted effects of immunomodulatory antibodies *in vivo* (22–24). As a direct correlate to human IgG4 does not exist in the mouse, evaluating the impact of the human IgG4 Fc-domain on antibody activity has to rely on *in vitro* experimental settings. Thus, it remains largely unknown if the activity of human immunomodulatory antibodies using the IgG4 format relies on the Fc-portion and if modulating the interaction of human IgG4 to human FcγRs may be a valid strategy to optimize antibody activity or, more importantly, may allow to limit unwanted side-effects.

To allow studying human IgG subclass activity *in vivo*, we have developed a humanized mouse model in which a human immune system is transplanted into immunodeficient mice. Additionally, use of mice lacking the expression of mouse activating FcγRs (NSG-FcRγ-/- mice) focusses the interaction of human antibodies injected into these animals to human FcγRs (23, 25–27). By applying the CD137-specific IgG4 antibody urelumab in a glycosylated and non-glycosylated variant we now show that IgG4 deglycosylation maintains the immunostimulatory activity of the antibody, demonstrated by expansion of peripheral blood T cells, but limits the infiltration of T cells into the liver. Interestingly, in addition to glycosylation, the dose of the antibody played a very important role in triggering a systemic cytokine release and organ toxicity, with lower doses having a more pronounced effect on cytokine release and organ toxicity compared to higher doses. Thus, our study emphasizes that complex mechanisms underlie human immunomodulatory antibody activity *in vivo* and that glycoengineering of supposedly inert IgG subclasses may be a valid option to improve antibody safety in humans.

## Results

### Expression of CD137 in humanized mice

The human IgG4 CD137-specific antibody urelumab has shown promising results in pre-clinical and clinical settings. It has also become clear, however, that liver toxicity and a cytokine release syndrome are major dose limiting factors in many patients (28). To allow studying human CD137-specific antibody activity *in vivo*, we made use of a modified humanized mouse model system allowing to study human antibody function in the context of a human immune system in the absence of mouse activating FcγRs through genetic ablation of the mouse common FcRγ-chain (NSG-FcRγ-/- mice) (23, 26, 27). We first analysed CD137 expression on human T cells, NK cells and monocytes present in these animals during the steady state. The gating strategy for human immune cells in humanized mice is depicted in **Figure S1**. As shown in **Figures S2A-C** low levels of CD137 were detectable on CD4^+^ and CD8^+^ T cells as well as on different NK and monocyte subsets in the peripheral blood, spleen, lymph node, bone marrow, and thymus of humanized mice and humans. The only immune cell subset expressing elevated levels of CD137 during the steady state, were small subsets of CD4 and CD8 double positive and double negative T cells in the peripheral blood of humanized mice and humans (**Figure S2A**). Thus, the humanized mouse model largely recapitulates CD137 expression patterns observed in humans.

### Impact of targeting CD137 with glycosylated and deglycosylated urelumab on body weight and temperature of humanized mice

**Figure 1:**
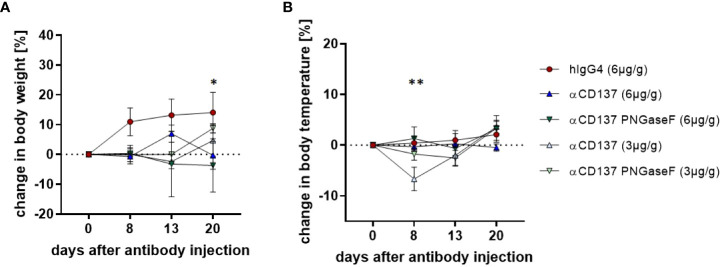
Effect of treatment with urelumab antibody variants on body weight and temperature in humanized mice. Shown are relative changes in body weight **(A)** and body temperature **(B)** of humanized mice after treatment with a human IgG4 isotype control (6µg/g) or 3µg/g or 6µg/g of urelumab (αCD137) or a deglycosylated urelumab variant (αCD137 PNGaseF) until twenty days after antibody injection (hIgG4: n=5, αCD137 6µg: n=7, αCD137 PNGaseF 6µg: n=3, αCD137 3µg: n=3, αCD137 PNGaseF 3µg: n=4). Shown is the mean+/-SEM of one representative out of two independent experiments. For assessment of statistical significance a Two Way ANOVA with Tukey’s multiple comparison test was used. In **(A)** * indicates p<0.05 for hIgG4 (6µg/g) vs. αCD137 (6µg/g) and hIgG4 (6µg/g) vs. αCD137 PNGaseF (6µg/g). In **(B)** ** indicates p<0.01 for hIgG4 (6µg/g) vs. αCD137 (3µg/g), αCD137 (6µg/g) vs. αCD137 3(µg/g) and αCD137 PNGaseF (6µg/g) vs. αCD137 (3µg/g).

To assess how urelumab impacts general health parameters, such as body weight and body temperature, we intravenously injected humanized mice once with 3 or 6µg/g body weight of urelumab or a human IgG4 isotype control antibody. Although human IgG4 antibodies are largely considered to have a low capacity to interact with cellular FcγRs or the complement system, more recent data clearly demonstrates that IgG4 in its monomeric form can bind to the high affinity FcγRI and that IgG4 immune complexes may bind to several activating FcγRs (22, 24). Importantly, deglycosylation of IgG4 was able to diminish this interaction, suggesting that glycan engineering may be an option to modulate human IgG4 effector functions *in vivo* (24). Thus, we also generated a deglycosylated urelumab variant by treating the parental antibody with PNGase F to assess if and to what extend IgG4 Fc-dependent effects played a role for urelumab activity *in vivo* in humanized mice. As expected, PNGaseF treatment resulted in a reduced molecular weight of urelumab and a complete loss of lens culinaris agglutinin (LCA) binding, which detects the mannose core of the N297-linked sugar moiety (**Figure S3A**). Further in line with our previous study deglycoylsation of urelumab resulted in a strongly reduced binding of urelumab to CHO cells expressing the human high affinity FcγRI (**Figure S3B**) (24). As shown in **Figure 1**, urelumab injection was tolerated well in general. Mice injected with both, the 3 or 6µg/g dose of the glycosylated and deglycosylated urelumab variants only showed transient changes in body temperature and a slight delay in gaining body weight compared to IgG4 isotype treated mice over the observation period of three weeks (**Figure 1A**). Interestingly, a major drop in body temperature occurred at the 3 (but not at the 6) µg/g dose of urelumab, while the deglycosylated urelumab variant showed a much milder and delayed reduction in body temperature (**Figure 1B**). Thus, we conclude that treatment with urelumab, especially the deglycosylated variant, is well tolerated by humanized mice.

### Effect of targeting CD137 with urelumab variants on human immune cells in humanized mice

**Figure 2:**
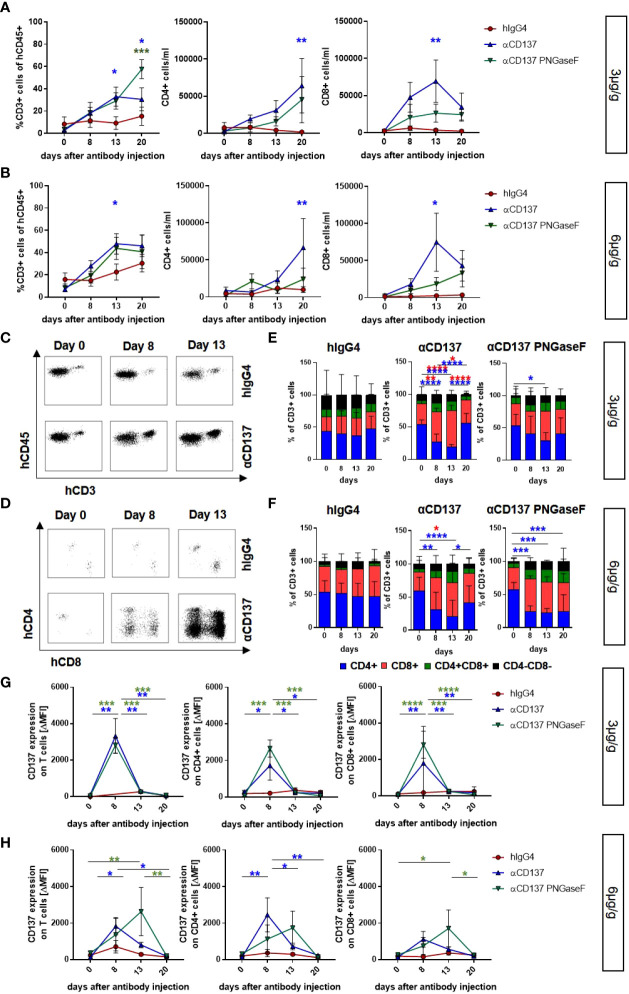
Impact of urelumab antibody dose and glycosylation on human T cells in the peripheral blood of humanized mice. Humanized mice received either 3 or 6µg/g of a human IgG4 isotype control, urelumab (αCD137) or deglycosylated urelumab (αCD137 PNGaseF) and were studied for the next twenty days after antibody injection. **(A, B)** Shown are the relative amounts of human CD3+ T cells (% of human CD45+ cells) and the absolute numbers of CD4+ or CD8+ T cells per ml blood of mice treated with 3µg/g (hIgG4: n= 5; αCD137: n=6; αCD137 PNGaseF: n=4) **(A)** or 6µg/g (hIgG4: n= 7-13; αCD137: n=10-11; αCD137 PNGaseF: n=6) **(B)** of the indicated antibody preparations. Coloured asterisks matching the respective colouring of the treatment group indicate significant differences at the indicated time-point of this group compared to the hIgG4 isotype control group. **(C-F)** Representative dot plots demonstrating the expansion of human CD3+ T cells **(C)** or CD4+ and CD8+ T cell subsets **(D)** in the peripheral blood of humanized mice before and at 8 or 13 days after injection of 6µg/g of the respective IgG4 antibodies; and quantification **(E, F)** of the relative abundance of human T cell subsets in mice receiving 3µg/g (hIgG4: n=6; αCD137: n=6 αCD137 PNGaseF: n=4) **(E)** or 6µg/g (hIgG4: n=11; αCD137: n=11 αCD137 PNGaseF: n=5) of the specified antibodies **(F)** at the indicated time points after injection. **(G, H)** CD137 expression (mean ΔMFI) on CD3+ (left panel), CD4+ (middle panel) and CD8+ (right panel) T cells in mice treated with 3µg/g **(G)** (hIgG4 n= 3; αCD137: n=6; αCD137 PNGaseF: n=4) or with 6µg/g **(H)** (hIgG4: n=7; αCD137: n=6-7; αCD137 PNGaseF: n=4-6) of the respective antibody preparations. Shown are pooled data from two to three independent experiments. Results are presented as mean +/-SEM. For statistical analysis 2-Way Anova with Tukey’s multiple comparison test was used to assess significant differences between experimental groups. *p<0.05; **p<0.01; ***p<0.001; ****p<0.0001.

**Figure 3:**
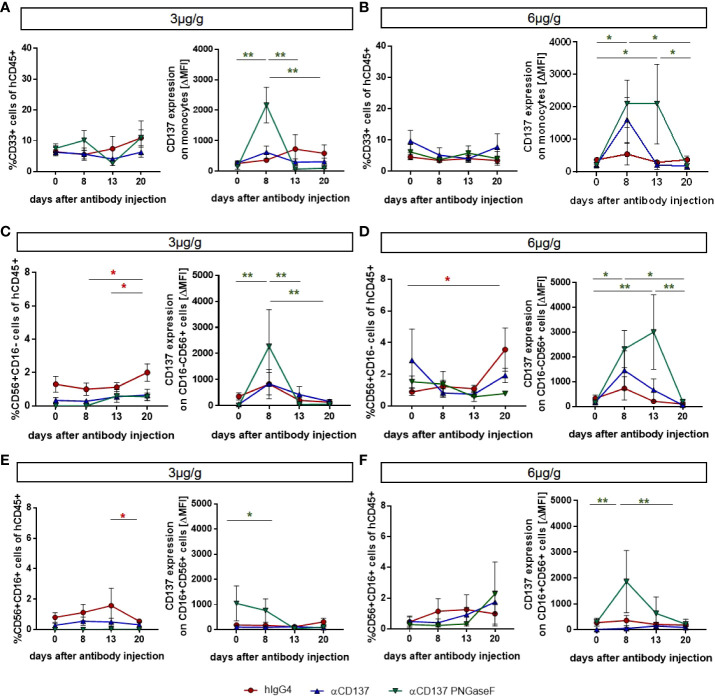
Effects of treatment with urelumab variants on human monocytes and NK cells in the peripheral blood of humanized mice. Humanized mice were injected with 3µg/g (hIgG4: n=3-4; αCD137: n=6; αCD137 PNGaseF: n=4) **(A, C, E)** or 6µg/g (hIgG4: n=7; αCD137: n=5; αCD137 PNGaseF: n=4) **(B, D, F)** of the human IgG4 isotype control, urelumab (αCD137), or deglycosylated urelumab (αCD137 PNGaseF) and studied for twenty days after antibody injection. **(A, B)** Shown are the relative changes in CD33+ monocyte numbers (left panel) as well as in CD137 expression (right panel, ΔMFI) on CD33+ monocytes at the indicated time-points after injection of 3 **(A)** or 6 **(B)** µg/g of the respective antibody preparations. **(C-F)** Shown are the relative changes in CD56^+^CD16^-^ **(C, D)** and CD56^+^CD16^+^ **(E, F)** NK cell subset abundance (left panel) as well as in CD137 expression (right panel) on the respective NK cell subsets at the indicated time-points after injection of 3 **(C, E)** or 6 **(D, F)** µg/g of the respective antibody preparations. Results are presented as mean +/-SEM. For statistical testing a 2-Way Anova with Tukey’s multiple comparison test was used. *p<0.05; **p<0.01.

**Figure 4:**
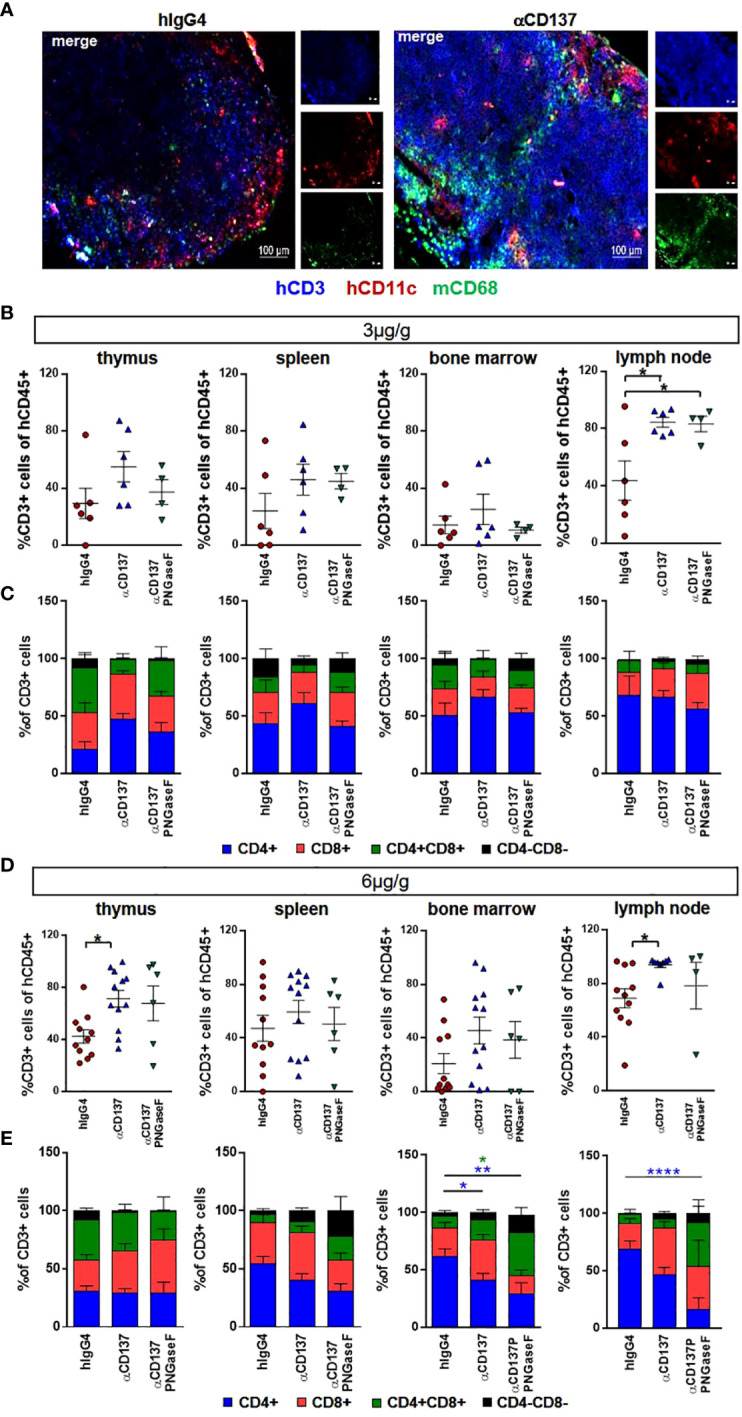
Effect of urelumab variant treatment on T cells in primary and secondary immunological organs. Humanized mice received either 3 or 6µg/g of a human IgG4 isotype control, urelumab (αCD137) or deglycosylated urelumab (αCD137 PNGaseF) and were studied for twenty days after antibody injection. **(A)** Shown are representative immunofluorescent stainings of lymph node sections of mice treated with 6µg/g hIgG4 as a control or with urelumab (αCD137) identifying human T cells (hCD3), human dendritic cells (hCD11c), as well as mouse macrophages (mCD68). **(B, C)** Depicted are relative amounts of human CD3+ cells **(B)** and of different T cell subsets **(C)** in thymus, spleen, bone marrow, and lymph nodes of mice treated with 3µg/g of the indicated antibodies (hIgG4: n= 6; αCD137: n=6; αCD137 PNGaseF: n=4). **(D, E)** Depicted are relative amounts of human CD3+ T cells **(D)** and of different T cell subsets **(E)** in thymus, spleen, bone marrow, and lymph nodes of mice treated with 6µg/g of the indicated antibody variants (hIgG4: n= 11; αCD137: n=8-12; αCD137-PNGaseF: n=4-6). Shown is the combined data from two to three independent experiments and results are represented as mean +/- SEM. Statistical analysis was done by Shapiro-Wilk normality test, Kruskal-Wallis with Dunn’s multiple comparison test or 1-way ANOVA. For analysing T cell subsets a 2-Way ANOVA was performed. *p<0.05; **p<0.01; ****p<0.0001.

In order to analyze the effect of urelumab variants on human T cell subsets in the peripheral blood of humanized mice, we performed flow cytometric analysis of immune cell subsets. We noted that both doses of urelumab triggered a strong expansion of both, CD4^+^ and CD8^+^ T cells (**Figures 2A, B**, **S4**). In line with observations in patients, urelumab injection triggered a faster and stronger expansion of CD8^+^ T cells starting at one and peaking at two weeks after urelumab injection, followed by a decline thereafter (**Figures 2A-F**). In contrast, a significant expansion of CD4^+^ T cells occurred roughly one week later and was preceded by a slow increase of CD4^+^ T cell numbers until day 13. While the deglycosylated urelumab variant showed a slower expansion of CD8^+^ T cells without the peak at day 13, the final number of CD8^+^ T cells at three weeks after antibody injection was the same. This was also evident for the expansion of CD4^+^ T cells at the 3µg/g dose, while the expansion of CD4+ T cells at the higher dose was diminished in animals receiving deglycosylated urelumab. With respect to relative changes in the different T cell subsets occurring after urelumab variant injection in humanized mice, CD8^+^ T cells showed the strongest expansion, followed by an increase in a small subset of CD4/CD8 double positive T cells (**Figures 2D, F**). While CD137 expression is low on T cells in the steady state, CD137 becomes upregulated upon T cell activation. Indeed, we observed a strong upregulation of CD137 on T cells eight days after injection of 3 or 6µg/g urelumab (**Figures 2G, H**). Of note, while no change in CD137 upregulation was observed in animals receiving the deglycosylated urelumab variant (αCD137 PNGase F) at the 3µg/g dose, a delayed upregulation on both, CD4^+^ and CD8^+^ T cells was noted at the 6µg/g dose. With respect to monocytes and NK cells, urelumab injection did not trigger changes in cell abundance at either antibody dose (**Figure 3**). Interestingly, however, the deglycosylated urelumab variant resulted in a stronger and prolonged upregulation of CD137 on NK cell subsets and monocytes, respectively (**Figures 3A-D, F**). Within lymphoid organs, both urelumab variants triggered a strong expansion of T cells in lymph nodes, whereas the effect on splenic T cells was much milder (**Figures 4A-C**). While the 3µg/g dose resulted in a more pronounced effect on CD4^+^ T cells in most organs, the 6µg/g dose induced a more dominant effect on CD8^+^ T cells (**Figures 4D, E**). Interestingly, the parental as well as the deglycosylated urelumab variant induced an expansion of CD8^+^ T cells in the thymus and lymph node at the 6µg/g dose (**Figure 4E**). Moreover, the deglycosylated variant of urelumab seemed to expand the small subset of CD4/CD8 double positive T cells in the lymph node and bone marrow of humanized mice (**Figure 4C**). In summary, our data indicate that deglycosylation of urelumab at most delays but does not impair T cell activation and expansion at both investigated antibody doses.

### Effect of urelumab glycosylation on serum cytokine levels

**Figure 5:**
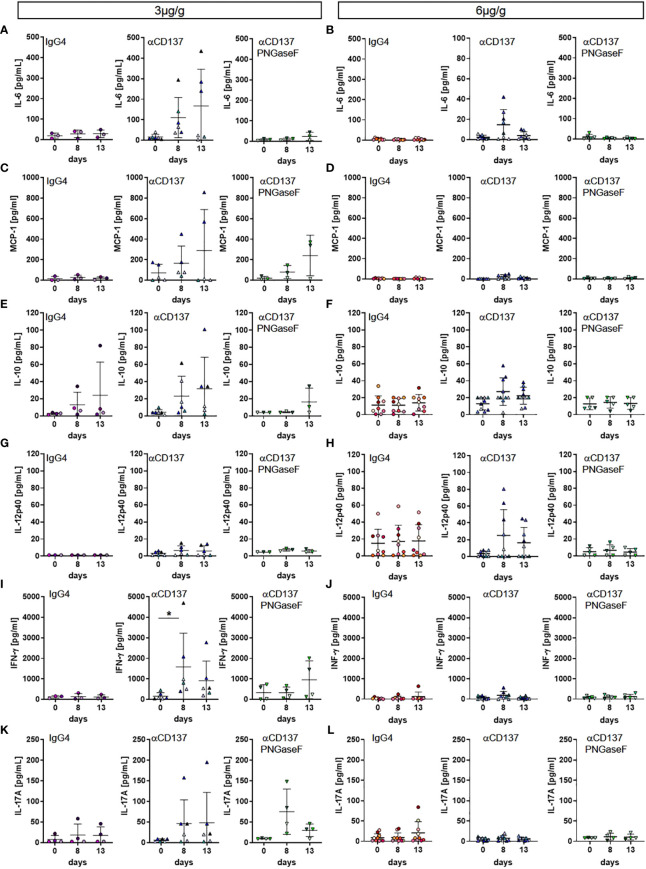
Effect of urelumab treatment dose and glycosylation on serum cytokine levels in humanized mice. Humanized mice received either 3 or 6µg/g of a human IgG4 isotype control, urelumab (αCD137) or deglycosylated urelumab (αCD137 PNGaseF) and serum samples were collected before and at 8 and 13 days after antibody injection. Shown are concentrations of serum cytokine levels before and at the indicated time-points after injection of 3 **(A, C, E, G, I, K)** or 6 **(B, D, F, H, J, L)** µg/g. Depicted are serum concentrations of IL-6 (hIgG4: n=3-8, αCD137: n=6-8, αCD137 PNGaseF: n=3-5) **(A, B)**, MCP-1 (hIgG4: n=3-7, αCD137: n=5-7, αCD137 PNGaseF: n=3-4) **(C, D)**, IL10 (hIgG4: n=4-9, αCD137: n=6-10, αCD137 PNGaseF: n=3-5) **(E, F)**, IL12p40 (hIgG4: n=3-9, αCD137: n=6-9, αCD137 PNGaseF: n=3-5) **(G, H)**, IFNγ (hIgG4: n=3-7, αCD137: n=6-8, αCD137 PNGaseF: n=4-5) **(I, J)**, and IL17A (hIgG4: n=4-8, αCD137: n=6-9, αCD137 PNGaseF: n=4) **(K, L)**. Results are expressed as mean +/-SD. Statistical analysis was done using ROUT outlier test (Q=1%) and Shapiro-Wilk normality test. For graphs showing serum concentration of cytokines either Friedman test with Dunn’s multiple comparison test or RM-One-Way ANOVA with Tukey’s multiple comparison test was performed. *p<0.05.

Major side effects potentially associated with the injection of CD137-specific antibodies are a cytokine release syndrome and/or immune cell infiltrations in organs such as the liver (8, 28). To assess if injection of the parental or the deglycosylated variant of urelumab induces an increase in human serum cytokine levels we quantified the serum levels of human IL1β, IL6, IL8, IL10, IL12p40, IL17a, IFNα, IFNγ, and MCP-1 eight and thirteen days after injection of 3 or 6µg/g of the respective antibody preparations. As shown in **Figures 5A, C, E, G, I, K**, **S5** we noted an increase of IL1β, IL6, IL8, MCP-1, IFNγ, and IL17a while IFNα, IL10 and IL12 levels increased only mildly or did not change upon injection of 3µg/g of urelumab. Deglycosylation of urelumab largely abrogated or greatly diminished serum cytokine levels, suggesting that IgG4 Fc-dependent effects were involved in triggering human cytokine release. Interestingly, injection of 6µg/g of urelumab trigger a much milder and transient cytokine release, which was fully abrogated upon deglycosylation of the antibody (**Figures 5B, D, F, H, J, L**, **S5** ). In summary, urelumab triggered cytokine release showed a clear dependence on a functional IgG4 Fc-domain.

### Effect of urelumab glycosylation on organ pathology

**Figure 6:**
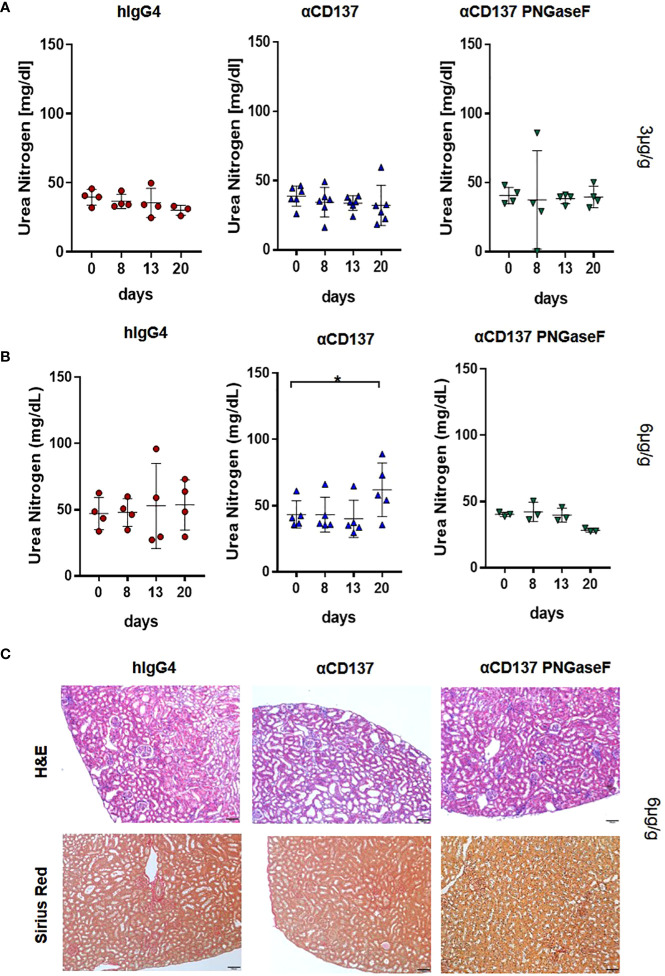
Impact of αCD137 treatment on kidney function. Humanized mice received either 3 or 6µg/g of a human IgG4 isotype control, urelumab (αCD137) or deglycosylated urelumab (αCD137 PNGaseF). **(A, B)** Shown are blood urea nitrogen (BUN) levels before (day 0) or at the indicated timepoints after treatment of humanized mice with 3 (hIgG4: n=4, αCD137: n=6, αCD137 PNGaseF: n=4) **(A)** or 6 (hIgG4: n=4, αCD137: n=5, αCD137 PNGaseF: n=3) **(B)** µg/g of the IgG4 antibody variants, as indicated. **(C)** Shown are hematoxylin-eosin (H–E) and Sirius red stainings of kidney sections of mice treated with 6µg/g of the indicated IgG4 antibody variants twenty days after antibody injection. Scale bars represent 100µm. Results are expressed as mean with SD. Statistical analysis was done by using Shapiro-Wilk normality test; p values were determined by using either Friedman test with Dunn’s multiple comparison analysis or One-Way-Anova with Tukey’s multiple comparison test. *p<0.05.

**Figure 7:**
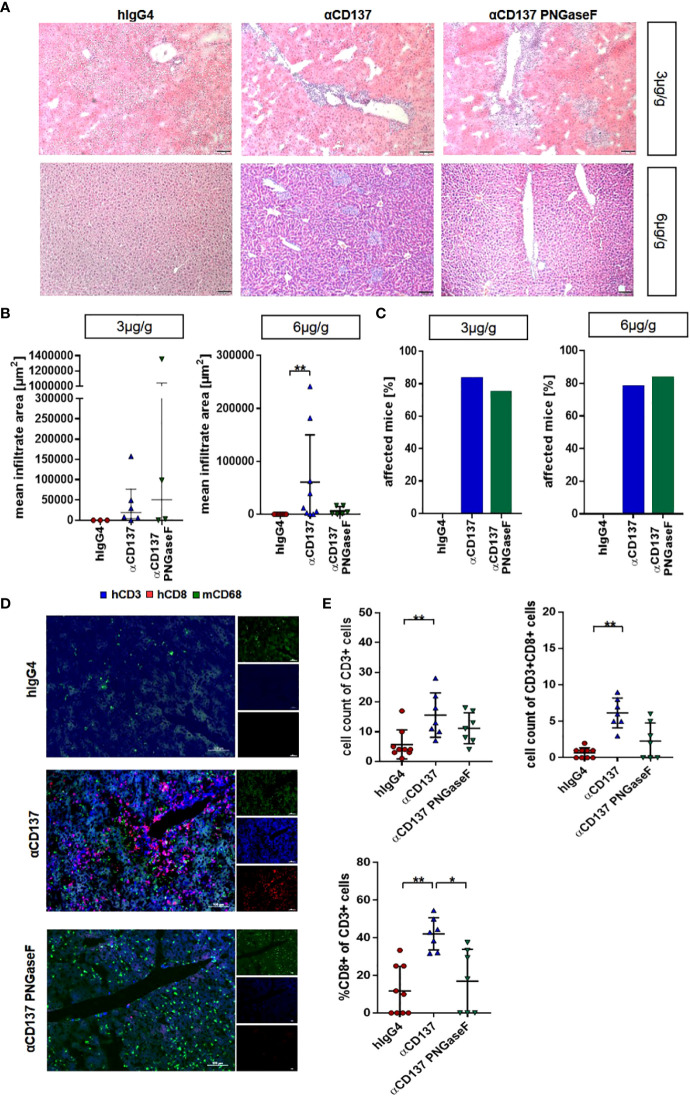
Impact of treatment with urelumab variants on liver pathology. Humanized mice received either 3 or 6µg/g of a human IgG4 isotype control, urelumab (αCD137) or deglycosylated urelumab (αCD137 PNGaseF) and liver pathology was studied twenty days after antibody injection. **(A, B)** Representative hematoxylin/eosin stained liver sections **(A)** of mice treated with 3 or 6µg/g of human IgG4 isotype control, urelumab and deglycosylated urelumab variants (scale bar 100µm) and quantification of the mean infiltrate area **(B)** of immune cell infiltrates (hIgG4: n=3, αCD137: n=6, αCD137 PNGaseF: n=4). **(C)** Shown is the percentage of mice with detectable immune cell infiltrates in the liver. **(D, E)** Immunofluorescent staining of liver sections (scale bar 100µm) **(D)** and quantification of the infiltration **(E)** of CD3+ T cells (upper panel) and the relative amount of CD3+CD8+ T cells within the CD3+ T cell population (lower panel) (hIgG4: n=9, αCD137: n=7, αCD137 PNGaseF: n=7, n represents the number of analysed images of two independent mice per group). Statistical testing was performed by using a Shapiro-Wilk and Kruskal Wallis test with a Dunn’s multiple comparison test. *p<0.05; **p<0.01.

As deglycosylated urelumab induced reduced cytokine levels, we next assessed whether IgG4 deglycosylation would also impact urelumab induced organ pathology. With respect to immune cell infiltrates into organs we focused on the kidney and liver (**Figures 6**, **7**). In the kidney, only mice receiving the 6µg/g dose showed slightly increased levels of blood urea nitrogen (BUN), indicative for a slightly impaired kidney functionality (**Figure 6A, B**). Deglycosylation of urelumab prevented this phenotype, again suggesting that IgG4 Fc-domain dependent effects played a role. With respect to kidney histology, no major immune cell infiltrates or major changes in glomerular structure were observed, further supporting the notion of a rather mild effect of urelumab on the kidney (**Figure 6C**). In contrast to the kidney, however, major immune cell infiltrates were noted in the liver at both antibody doses (**Figures 7A-C**). Whereas urelumab deglycosylation did not reduce the number of mice showing immune cell infiltrations (**Figure 6C**), injection of deglycosylated urelumab resulted in a greatly diminished size of immune cell infiltrations at the 6µg/g dose (**Figure 6B**). In contrast, no major effect of urelumab deglycosylation on immune cell infiltration into the liver became visible at the 3µg/g dose. A more detailed analysis of the immune cell infiltrations of animals receiving the 6µg/g urelumab dose by immunofluorescence analysis further revealed that T cells were a major component of the immune cell infiltrates and that urelumab deglycosylation reduced the number of cytotoxic T cells within the liver (**Figures 7D, E**). In summary, our results suggest that urelumab deglycosylation diminishes the release of pro-inflammatory cytokines at both antibody doses, while an infiltration of the liver by T cells could only be reduced at the 6µg/g dose.

## Discussion

The introduction of checkpoint control and immunomodulatory antibodies in the therapy of cancer, autoimmune diseases or after solid organ transplantation marks a new era in immunotherapy. One agonistic antibody target that showed very promising results in pre-clinical studies in mouse models of cancer and autoimmunity is CD137, which is mainly expressed on activated T cells, monocytes, and NK cells (29–31). In humans two different CD137-specific antibodies, the IgG4 antibody urelumab or the IgG2 antibody utomilumab were tested in clinical trials (8, 28). Both antibody formats were chosen to limit unwanted side-effects *via* the interaction of the antibody Fc-domain with cellular FcγRs and the complement pathway. However, while the IgG4 antibody urelumab was characterized by a higher agonistic activity compared to utomilumab, it also triggered more severe side-effects, including a systemic cytokine release syndrome and hepatotoxicity requiring to readjust antibody dosing to levels at which clinical trials using urelumab as a monotherapy showed disappointing results (28). Despite the notion that IgG4 Fc-domains may not play a role in mediating urelumab activity, we reasoned that IgG4 immune complexes, formed *in vivo* upon CD137-specific antibody binding to T cells for example, may productively interact with human FcγRs and trigger Fc-dependent activities as suggested by several previous reports (22–24). Aiming to analyse this *in vivo* in the setting of a human immune system, we used deglycosylated IgG4, which has a strongly reduced capacity to bind to cellular FcγRs; and chose our well established NSG-FcRγ mouse model. This allows the transplantation of a human immune system by injection of human hematopoietic stem cells into immunodeficient mice, lacking all mouse activating FcγRs, at the day of birth (23, 27, 32).

Consistent with a model in which the severe side effects triggered by urelumab injection are mediated by the interaction of the IgG4 Fc-domain with cellular FcγRs, our study demonstrates that a deglycosylated IgG4 variant of urelumab, which has a strongly reduced ability to bind cellular FcγRs, loses its ability to trigger a strong, systemic release of pro-inflammatory cytokines at both antibody doses. In addition, deglycosylated urelumab failed to recruit immune cell infiltrates into the liver at the high (but not at the low) antibody dose and did not trigger increased blood urea nitrogen levels. In contrast, only a mild reduction or delay in T cell proliferation was noted, suggesting that at least many of the beneficial immunomodulatory effects mediated by urelumab were maintained in the absence of urelumab glycosylation. Our study further suggests, that a much stronger and qualitatively different cytokine release was observed at the 3µg/g dosing scheme, which correlated with a drop in body temperature observed eight days after injection of urelumab. In contrast, deglycosylated urelumab injection resulted only in a minor and delayed reduction in body temperature. The observation that higher doses of urelumab may result in diminished release of pro-inflammatory cytokines is consistent with a recent study by Qi and colleagues demonstrating that human T cells secret less IFNγ when stimulated with 1 instead 0.3µg/ml of urelumab *in vitro* in the presence of FcγR expressing cells (21). The phenomenon that higher IgG doses may trigger less FcγR-dependent effects compared to lower antibody doses is well known as the Heidelberger-Kendall curve, although a more detailed titration would be necessary to demonstrate this more convincingly in our study (33, 34).

One surprising finding of our study was that urelumab deglycosylation strongly impairs immune cell infiltration into the liver at the 6µg/g dose but not at the 3µg/g antibody dose. Again, the Heidelberger-Kendall curve would predict that larger immune complexes could be formed at a lower antibody dose, which may retain binding to at least some activating FcγRs, such as FcγRI, even in an aglycosylated form as shown in this and our previous studies (24). As no FcγRI-specific antibody with highly efficient blocking activity for IgG binding is available, it is currently not possible to test this hypothesis directly. An alternative explanation may be afforded by the much more pronounced cytokine release syndrome observed at the lower antibody dose, which may trigger an infiltration of immune cells into the liver. However, urelumab deglycosylation largely blunted this cytokine release, yet the immune cell infiltration in the liver was maintained. Thus, this result rather suggests, that the cytokine release can be uncoupled from immune cell infiltration into the liver. More studies will be necessary to fully unravel the molecular and cellular basis for this result.

With respect to the need for a productive interaction of the Fc-domain of CD137-specific antibodies for stimulating T cell proliferation, several studies in mice demonstrated that different CD137-specific antibodies required a functional Fc-domain for maintaining their T cell stimulatory activity *in vivo* (10, 20, 21). The results of our study suggest that urelumab-dependent T cell proliferation may be maintained in the absence of an FcγR engaging Fc-domain, while unwanted side effects including the systemic cytokine release can be reduced. A major limitation of our study at present is that this humanized mouse model does not allow to assess the therapeutic activity of deglycosylated urelumab in the setting of tumor or autoimmune disease, for example. Without matching the tumor MHC haplotype to the human immune system it would be difficult to assign a reduction in tumor growth to the enhancement of tumor-specific immune reactions. Nonetheless, the maintenance of T cell proliferation upon injection of deglycosylated urelumab may indicate that the effect of T cell expansion can be uncoupled from the unwanted side-effects and hence may be less dependent on a functional Fc-domain.

## Experimental procedures

### Human material

Human material (blood, spleen, bone marrow and thymus samples) was provided by the University Hospital Erlangen. Leukocyte reduction cones were obtained from anonymous healthy adult donors, thymus samples were derived from cardiac surgeries of healthy children, spleen samples were collected from patients requiring therapeutic splenectomy, and bone marrow was obtained from biopsies performed to exclude bone marrow involvement in cancer. All samples were obtained under local ethical committee approvals (Ethikkommission der Friedrich-Alexander-Universität Erlangen-Nürnberg), and informed written consents were obtained in accordance with the Declaration of Helsinki. In brief, thymic and splenic tissues were chopped into small pieces using forceps and scalpel. The tissue was transferred into C-tubes (Miltenyi Biotec), filled with 5 ml RPMI1640, further mechanically disrupted using a Gentle MACS tissue dissociator (Miltenyi Biotec), and enzymatically digested with 400 U/ml collagenase D (Serva) and 100 μg (spleen) or 300 μg (thymus) deoxyribonuclease I (Sigma). After filtering the cell suspension twice, cell suspension of splenic and thymic tissue as well as the leukocyte enriched fraction of human blood was diluted with RPMI1640 and a density gradient centrifugation using Human Pancoll (ρ = 1.077 g/ml; Pan Biotech) was performed as described earlier. Bone marrow was filtered using a 100 µm cell strainer prior to the density gradient centrifugation. After the centrifugation, the interphase containing the mononuclear cells was collected, washed twice with RPMI1640, and used for experiments or resuspended in FCS + 10% DMSO at a final concentration of 5*10^7^ cells/ml and stored at -80 °C until analysis.

### Mice

FcRγ-/- mice, deficient for the fcer1 gene, were provided by Jeffery Ravetch (Rockefeller University, New York, USA), whereas NOD-, SCID-, and γc-deficient mice were supplied by the Jackson Laboratories. For generating NOD-SCID/γc/FcRγ-/- (NSG-FcRγ-/-) mice the γc-/- as well as fcer1g-/- mouse strains were back-crossed to the NOD/Scid background for at least ten generations. Mice were kept according to the guidelines of the National Institutes of Health and the legal requirements of Germany.

### Generation of humanized mice

The humanization of NSG-FcRγ-/- mice was performed as described before (35). In brief, hematopoietic stem cells were isolated from human umbilical cord blood using a ‘Direct CD34 Progenitor Cell Isolation Kit, human’ (Miltenyi Biotec) according to the manufacturer’s instructions and frozen and stored in liquid nitrogen until further use. New-born NSG-FcRγ-/- mice were irradiated with a dose of 1.4 Gy within the first 24 hours after birth. 6-18 hours after irradiation, hematopoietic stem cells were injected intravenously into the facial vein (20,000-50,000 HSCs). Peripheral blood of transplanted mice was analyzed at 10-12 weeks of age and mice having greater than 5% hCD45+ cells in the peripheral blood were arbitrarily allocated to experimental groups and used for further experiments. As far as possible different humanized mice generated from one HSC donor received different treatments (isotype control, unmodified or aglycosylated urelumab) to allow a better comparison between control and experimental groups. With respect to comparison between 3 and 6µg/g treatment groups we aimed at including as many HSC donors as possible to limit batch effects of individual HSC donors. Thus, we included humanized mice generated from 22 different HSC donors in the experiments to mimic a diverse human clinical situation.

### Antibody injection

Urelumab was injected intravenously at a dose of 3 or 6µg/g. As an isotype control, a human IgG4 antibody was administered. In addition, a deglycosylated variant of urelumab was generated by treating urelumab with 10 Units per µg IgG PNGase F (NEW ENGLAND BioLabs, Cat.#: P0704L) over night at 37°C. Urelumab deglycosylation was verified using lectin blot analysis with Lens culinaris agglutinin as described before (36)

### Flow cytometric analysis

Peripheral blood (100μL) was collected by retro orbital puncture before and at select time points after antibody treatment. Single cell preparations from organs were generated by using a 70µm cell strainer. Erythrocytes were lysed using ddH_2_O followed by adding 10x PBS to stop the lysis. After washing, cells were re-suspended in Fc-Block (0.5 μg/well, 2.4G2) and incubated for 15 min on ice, followed by another washing step and staining for 15 min at 4°C with fluorochrome-conjugated antibodies (see **Table 1**). Finally, DAPI was added (dilution 1:5,000) for identifying dead cells, cells were washed again, and re-suspended in 100 μl FACS buffer followed by analysis on a FACS Canto II. Data was evaluated using the BD Diva or FlowJo software.

**Table 1:** Key reagents used for the study.

Reagent type (species) or resource	Designation	Source reference	Catalogue number	Analysis and dilution
**Antibody**	Anti-human CD3 PerCP (mouse monoclonal) Clone UCHT1	BioLegend	Cat.#: 300428	Flow cytometry (1:200)
**Antibody**	Anti-human CD3 Brilliant Violet 510™ (mouse monoclonal) Clone SK7	BioLegend	Cat.#: 344828	Flow cytometry (1:100)
**Antibody**	Anti-human CD3 Alexa Fluor^®^ 647 (mouse monoclonal) Clone UCHT1	BioLegend	Cat.#: 300416	Immuno- histochemistry (1:20)
**Antibody**	Anti-human CD4 FITC (mouse monoclonal) Clone IV T114	BioLegend	Cat.#: 300506	Flow cytometry (1:200)
**Antibody**	Anti-human CD8 PE (mouse monoclonal) Clone SK1	BioLegend	Cat.#: 344706	Immuno- histochemistry (1:20)
**Antibody**	Anti-human CD8 PE/Cy7 (mouse monoclonal) Clone SK1	BioLegend	Cat.#: 344712	Flow cytometry (1:200)
**Antibody**	Anti-human CD14 PE (mouse monoclonal) Clone M5E2	BioLegend	Cat.#: 301806	Flow cytometry (1:50)
**Antibody**	Anti-human CD16 FITC (mouse monoclonal) Clone 3G8	BioLegend	Cat.#: 302006	Flow cytometry (1:100)
**Antibody**	Anti-human CD33 Brilliant Violet 510™ (mouse monoclonal) Clone WM53	BioLegend	Cat.#: 303422	Flow cytometry (1:100)
**Antibody**	Anti-human CD45 APC/Fire™ 750 (monoclonal mouse) Clone HI30	BioLegend	Cat.#: 304062	Flow cytometry (1:200)
**Antibody**	Anti-mouse CD45.1 Brilliant Violet 421™ (mouse monoclonal) Clone A20	BioLegend	Cat.#: 110732	Flow cytometry (1:600)
**Antibody**	Anti-human CD56 PE/Cy7 (mouse monoclonal) Clone MEM-188	BioLegend	Cat.#: 304628	Flow cytometry (1:100)
**Antibody**	Anti-mouse CD68 Alexa Flour^®^ 488 (rat monoclonal) Clone FA-11	BioLegend	Cat.#: 137011	Immuno- histochemistry (1:50)
**Antibody**	Anti-human CD137 APC (mouse monoclonal) Clone 4B4-1	BioLegend	Cat.#: 309810	FACS (1:100)
**Antibody**	Urelumab (αCD137)	In house (UK Erlangen)	—	i.v. injection
**Antibody**	Urelumab PNGase F digested	Digestion in house		i.v. injection
**Antibody**	IgG4 (S228P) isotype control	BioXcell	Cat.#: CP147	i.v. injection
**Kit**	LegendPlex™ Multi-Analyte Flow Assay Kit Custom Human Assay	BioLegend^®^	info@biolegend.com	Cytokine detection
**Enzyme**	PNGase F	NEW ENGLAND BioLabs	Cat.#: P0704L	Antibody digestion
**Kit**	Urea Nitrogen (BUN) Reagent Set (Colorimetric Method)	TECO DIAGNOSTICS	Cat.#: B551-132	Blood Urea Nitrogen
**Kit**	CD34 Micro Bead Kit, human	Miltenyi Biotec	Cat.#: 130-046-702	Stem cell isolation

### Binding of urelumab variants to human FcγRI

100000 CHO cells or CHO cells expressing FcγRI were incubated with either 1µg of untreated urelumab (αCD137), 1µg of PNGase F treated urelumab (αCD137-PNGase F) or with PBS in 100µl FACS buffer for 1h on ice. After incubation, cells were washed and stained for 15 min on ice with either Protein L PE (1:10) in FACS buffer. Cells were washed again and resuspended in 50µL of FACS buffer and analysed at FACS CANTO II.

### Cytokine release assay

For the determination of human cytokine levels, peripheral blood was obtained (as described above), incubated for 30min. at RT, and centrifuged at 10,000xg for 5 min. Serum was collected by taking off the supernatant, which was stored at -80°C until further use. To measure human cytokine levels in the serum, the human LEGENDplex™ Multi-Analyte Flow Assay Kit (Biolegend) was used according to the instructions of the manufacturer. Samples were analyzed by FACS analysis on a FACS Canto II and the data was analyzed by using Biolegend’s LEGENDplex™ Data Analysis Software.

### Determination of kidney dysfunction

For monitoring kidney dysfunction, blood urea nitrogen was measured using urea nitrogen colorimetric kit (Teco Diagnostics) using one tenth of the recommended volume. 0.5 µL serum was used and mixed with the BUN Enzyme Reagent. After 10 min of incubation at RT, Bun Color developer was added and again incubated 10 min at RT. The absorbance at 570nm was measured with ‘VersaMax tunable microplate reader’ (Molecular Devices).

### Immunohistochemistry

Organ samples were frozen at -80°C in OCT and cut into 5 μM sections followed by fixation for 2.5 minutes with acetone. Unspecific antibody binding was prevented by incubation of the sections with blocking solution (5% goat serum in PBS) for 1 hour at RT. Blocking solution was removed and fluorochrome-conjugated antibodies were added for staining in 5% goat serum in PBS and incubated for 30 min at RT in the dark. Slides were rinsed 3 times with 1xPBS, mounted with a drop of mounting medium and dried for 30 minutes. Stained sections of liver and kidney were analyzed on an Axiovert 200M microscope.

For determination of liver and kidney pathology, Sirius Red collagen staining was performed to detect morphological abnormalities. H&E staining of liver and kidney samples was used for determination of cell-infiltrates. For both stainings, liver and kidney samples were embedded in paraffin and cut into 5 µM sections. Stainings were done using standard protocols beginning with paraffin removal using xylene, isopropanol and 96% ethanol. H&E staining was performed by incubating the sections for 8 min in Mayer’s hematoxylin solution (Merck 1:5), followed by washing steps and a 1 min incubation in eosin solution (Roth). After another washing step, slides were dipped into 96% ethanol, isopropanol and xylene. A drop of ROTI^®^ Histokitt II ready-to use-solution (Roth) was placed over the tissue on each slide and a coverslip was added. For Sirius Red staining the slides were treated for 6 min with Weigert’s iron hematoxylin (Roth), washed and then incubated in picro-sirius-red-solution for 5 min. Afterwards the sections were dipped in 100% ethanol and xylene. The sections were covered with a drop of ROTI^®^ Histokitt II ready-to use-solution (Roth) and a coverslip was placed above the tissue.

For further examination of liver damage, H&E stained liver sections were examined and liver immune-cell-infiltrates measured using CellSens 1.14 Software (OLYMPUS). Cell-infiltrates were marked and their area was calculated automatically. To determine the mean infiltrate size, three images of a liver section were analyzed per mouse. For a more detailed examination of cell types infiltrating the liver, immunofluorescent staining of liver sections was performed. Human T cells were stained by CD3 and CD8 was used for human cytotoxic T cells (see **Table 1**). The overall T cell count and the CD8^+^ T cell count was determined by counting the cells on 7-9 liver sections per group.

### Statistical analysis

GraphPad Prism 7.03 software (GraphPad Software Inc, Sand Diego, CA) was used for graphs and statistical analysis. Data are given in means ± standard error of the mean (SEM) or standard deviation (SD). All samples were tested for Gaussian distribution. Dependent on the Shapiro-Wilk normality test and on the comparative data we used one-way or two-way analysis of variance (ANOVA), Friedman or Kruskal-Wallis-test followed by a multiple comparison test (Tukey or Dunn). Data was further evaluated by using ROUT’s Outlier test (Q=1%). Detailed information of statistical test of individual results can be found in respective figure legends.

## Data availability statement

The raw data supporting the conclusions of this article will be made available by the authors, without undue reservation.

## Ethics statement

The animal study was reviewed and approved by Government of Lower Franconia.

## Author contributions

CR, AI-E, AL, CH and LH performed experiments, analyzed the data, and wrote the manuscript. DD contributed essential reagents. FN wrote the manuscript. All authors contributed to the article and approved the submitted version.

## Funding

This study was funded by the Deutsche Forschungsgemeinschaft (DFG, German Research Foundation - SFB TRR 305 - B02 to FN SFB TRR 305 - B05 to DD, and DU548/5-1 to DD), and by the Emerging Fields Initiative Big-Thera of the FAU (to FN and DD).

## Acknowledgments

We are grateful to Heike Albert and Heike Danzer for expert technical assistance and to Robert Cesnjevar for providing human tissue samples.

## Conflict of interest

The authors declare that the research was conducted in the absence of any commercial or financial relationships that could be construed as a potential conflict of interest.

## Publisher’s note

All claims expressed in this article are solely those of the authors and do not necessarily represent those of their affiliated organizations, or those of the publisher, the editors and the reviewers. Any product that may be evaluated in this article, or claim that may be made by its manufacturer, is not guaranteed or endorsed by the publisher.

## Supplementary material

Supplementary Figure 1 Identification of human immune cell subsets in humanized mice *via* flow cytometry. Shown is an exemplary gating strategy separating mouse immune cells (mCD45+) from human immune cells (hCD45+) in splenic single cell preparations of humanized mice three months after HSC transplantation. Human immune cell subsets (hCD45+) were identified by expression of human immune cell lineage specific markers, such as CD3, CD4, and CD8 to detect human CD8+ and CD4+ T cell subsets or CD56 or CD33 to identify natural killer (NK) cells or monocytes, respectively. CD16 in combination with CD56, as well as CD16/CD14 in combination with CD33 was used to identify further subsets of NK cells or monocytes.

Supplementary Figure 2 Comparison of CD137 expression on human T cells, NK cells and monocytes in humanized mice and humans. **(A)** Shown is CD137 expression on CD3^+^ T cell subsets in humanized mice in blood (n=37), spleen, bone marrow, thymus and lymph node (n=8) samples. **(B)** Shown is CD137 expression on the indicated human CD3^+^ T cell subsets in human peripheral blood, spleen, bone marrow as well as on thymic T cells (n=3). **(C, D)** Shown is CD137 expression on NK cell subsets in the respective organs in humanized mice (blood (n=33), spleen and bone marrow (n= 12)) **(C)** and humans (n=3) **(D)**. **(E, F)** Depicted is CD137 expression on CD33^+^ monocyte subsets located within the indicated organs in humanized mice (blood: n=33, spleen and bone marrow: n= 12) **(E)**, as well as in the respective human organs **(F)** (n=3). Results are expressed as median of ΔMFI with interquartile range.

Supplementary Figure 3 Effect of urelumab deglycosylation on binding to human FcγRI. **(A)** Shown is a Coomassie stained PAA gel (left panel) and a western blot analysis (right panel) using lens culinaris agglutinin (LCA) to detect the effect of deglycosylation on urelumab size or the urelumab core sugar structure, respectively. **(B)** Depicted is the binding of untreated urelumab (αCD137) or of PNGaseF treated urelumab (αCD137 PNGaseF) to CHO cells (CHO-Blank) or CHO cells expressing human FcγRI (CHO-FcγRI) as the delta mean fluorescence intensity (ΔMFI) as determined by flow cytometry. Bound urelumab was detected with fluorescently labelled protein L. As a further control, CHO cells as well as CHO-FcγRI expressing cells were only stained with protein L in the absence of urelumab variants (∅). Shown is the mean +/- SEM of two independent experiments.

Supplementary Figure 4 Effect of urelumab variants on T cells in the peripheral blood. Shown are the absolute numbers of CD3+ human T cells in the peripheral blood of humanized mice upon treatment with 3µg/g (hIgG4: n= 5; αCD137: n=6; αCD137 PNGaseF: n=4) or 6µg/g (hIgG4: n= 7-13; αCD137: n=10-11; αCD137 PNGaseF: n=6) of a human IgG4 isotype control antibody, urelumab (αCD137) or a PNGaseF treated urelumab variant (αCD137 PNGaseF). For each time point the mean+/-SEM is shown.

Supplementary Figure 5 Effect of urelumab variant injection on serum cytokine levels. Serum cytokine concentrations were determined before and at the indicated time-points after treating humanized mice with 3µg/g **(A, C, E)** or 6µg/g **(B, D, F)** of hIgG4, αCD137 or deglycosylated αCD137 PNGaseF variant. Depicted are serum concentrations of **(A)** IL-1β (hIgG4: n=3, aCD137: n=6, αCD137 PNGaseF: n=4), **(C)** IL-8 (hIgG4: n=3, αCD137: n=6, αCD137 PNGaseF: n=4), and **(E)** IFN-α (hIgG4: n=4, αCD137: n=6, aCD137 PNGaseF: n=4) in mice treated with 3µg/g of the indicated antibody variants. Concentration of **(B)** IL-1β (hIgG4: n=7, aCD137: n=9, aCD137 PNGaseF: n=5), **(D)** IL-8 (hIgG4: n=8, αCD137: n=8, αCD137 PNGaseF: n=4), and **(F)** IFN-α (hIgG4: n=9, αCD137: n=8, αCD137 PNGaseF: n=5), in mice treated with 6µg/g of antibody variants. Results are expressed as median of ΔMFI with interquartile range. A ROUT outlier test (Q=1%) and Shapiro-Wilk normality test were performed followed by either Friedman test with Dunn’s multiple comparison test or RM-One-Way ANOVA with Tukey’s multiple comparison test was performed.

## References

[1] Kaplon H Reichert JM. Antibodies to watch in 2019. MAbs (2019) 11:219–38. doi: 10.1080/19420862.2018.1556465 PMC6380461 30516432

[2] Demaria O Cornen S Daeron M Morel Y Medzhitov R Vivier E. Harnessing innate immunity in cancer therapy. Nature (2019) 574:45–56. doi: 10.1038/s41586-019-1593-5 31578484

[3] Ribas A Wolchok JD. Cancer immunotherapy using checkpoint blockade. Science (2018) 359:1350–5. doi: 10.1126/science.aar4060 PMC7391259 29567705

[4] Paluch C Santos AM Anzilotti C Cornall RJ Davis SJ. Immune checkpoints as therapeutic targets in autoimmunity. Front Immunol (2018) 9:2306. doi: 10.3389/fimmu.2018.02306 30349540 PMC6186808

[5] Sun Y Chen JH Fu Y. Immunotherapy with agonistic anti-CD137: two sides of a coin. Cell Mol Immunol (2004) 1:31–6. 16212918

[6] Sun Y Lin X Chen HM Wu Q Subudhi SK Chen L. Administration of agonistic anti-4-1BB monoclonal antibody leads to the amelioration of experimental autoimmune encephalomyelitis. J Immunol (2002) 168:1457–65. doi: 10.4049/jimmunol.168.3.1457 11801689

[7] Hassel JC Heinzerling L Aberle J Bahr O Eigentler TK Grimm MO. Combined immune checkpoint blockade (anti-PD-1/anti-CTLA-4): Evaluation and management of adverse drug reactions. Cancer Treat Rev (2017) 57:36–49. doi: 10.1016/j.ctrv.2017.05.003 28550712

[8] Segal NH Logan TF Hodi FS McDermott D Melero I Hamid O. Results from an integrated safety analysis of urelumab, an agonist anti-CD137 monoclonal antibody. Clin Cancer Res (2017) 23:1929–36. doi: 10.1158/1078-0432.CCR-16-1272 27756788

[9] Knorr DA Dahan R Ravetch JV. Toxicity of an fc-engineered anti-CD40 antibody is abrogated by intratumoral injection and results in durable antitumor immunity. Proc Natl Acad Sci U.S.A. (2018) 115:11048–53. doi: 10.1073/pnas.1810566115 PMC6205479 30297432

[10] Buchan SL Dou L Remer M Booth SG Dunn SN Lai C. Antibodies to costimulatory receptor 4-1BB enhance anti-tumor immunity *via* T regulatory cell depletion and promotion of CD8 T cell effector function. Immunity (2018) 49:958–970 e957. doi: 10.1016/j.immuni.2018.09.014 30446386

[11] Bulliard Y Jolicoeur R Windman M Rue SM Ettenberg S Knee DA. Activating fc gamma receptors contribute to the antitumor activities of immunoregulatory receptor-targeting antibodies. J Exp Med (2013) 210:1685–93. doi: 10.1084/jem.20130573 PMC3754864 23897982

[12] Coe D Begom S Addey C White M Dyson J Chai JG. Depletion of regulatory T cells by anti-GITR mAb as a novel mechanism for cancer immunotherapy. Cancer Immunol Immunother (2010) 59:1367–77. doi: 10.1007/s00262-010-0866-5 PMC11030908 20480365

[13] Marabelle A Kohrt H Sagiv-Barfi I Ajami B Axtell RC Zhou G. Depleting tumor-specific tregs at a single site eradicates disseminated tumors. J Clin Invest (2013) 123:2447–63. doi: 10.1172/JCI64859 PMC3668834 23728179

[14] Simpson TR Li F Montalvo-Ortiz W Sepulveda MA Bergerhoff K Arce F. Fc-dependent depletion of tumor-infiltrating regulatory T cells co-defines the efficacy of anti-CTLA-4 therapy against melanoma. J Exp Med (2013) 210:1695–710. doi: 10.1084/jem.20130579 PMC3754863 23897981

[15] Li F Ravetch JV. Inhibitory fcgamma receptor engagement drives adjuvant and anti-tumor activities of agonistic CD40 antibodies. Science (2011) 333:1030–4. doi: 10.1126/science.1206954 PMC3164589 21852502

[16] White AL Chan HT Roghanian A French RR Mockridge CI Tutt AL. Interaction with FcgammaRIIB is critical for the agonistic activity of anti-CD40 monoclonal antibody. J Immunol (2011) 187:1754–63. doi: 10.4049/jimmunol.1101135 21742972

[17] Lux A Nimmerjahn F. No need for constant help: human IgG2 antibodies have an autonomous agonistic activity for immunotherapy of cancer. Cancer Cell (2015) 27:10–1. doi: 10.1016/j.ccell.2014.12.010 25584890

[18] White AL Chan HT French RR Willoughby J Mockridge CI Roghanian A. Conformation of the human immunoglobulin g2 hinge imparts superagonistic properties to immunostimulatory anticancer antibodies. Cancer Cell (2015) 27:138–48. doi: 10.1016/j.ccell.2014.11.001 PMC4297290 25500122

[19] Dahan R Sega E Engelhardt J Selby M Korman AJ Ravetch JV. FcgammaRs modulate the anti-tumor activity of antibodies targeting the PD-1/PD-L1 axis. Cancer Cell (2015) 28:543. doi: 10.1016/j.ccell.2015.09.011 28854351

[20] Ho SK Xu Z Thakur A Fox M Tan SS DiGiammarino E. Epitope and fc-mediated cross-linking, but not high affinity, are critical for antitumor activity of CD137 agonist antibody with reduced liver toxicity. Mol Cancer Ther (2020) 19:1040–51. doi: 10.1158/1535-7163.MCT-19-0608 31974274

[21] Qi X Li F Wu Y Cheng C Han P Wang J. Optimization of 4-1BB antibody for cancer immunotherapy by balancing agonistic strength with FcgammaR affinity. Nat Commun (2019) 10:2141. doi: 10.1038/s41467-019-10088-1 31105267 PMC6526162

[22] Bruhns P Iannascoli B England P Mancardi DA Fernandez N Jorieux S. Specificity and affinity of human fcgamma receptors and their polymorphic variants for human IgG subclasses. Blood (2009) 113:3716–25. doi: 10.1182/blood-2008-09-179754 19018092

[23] Lux A Seeling M Baerenwaldt A Lehmann B Schwab I Repp R. A humanized mouse identifies the bone marrow as a niche with low therapeutic IgG activity. Cell Rep (2014) 7:236–48. doi: 10.1016/j.celrep.2014.02.041 24685130

[24] Lux A Yu X Scanlan CN Nimmerjahn F. Impact of immune complex size and glycosylation on IgG binding to human FcgammaRs. J Immunol (2013) 190:4315–23. doi: 10.4049/jimmunol.1200501 23509345

[25] Brandsma AM Hogarth PM Nimmerjahn F Leusen JH. Clarifying the confusion between cytokine and fc receptor “Common gamma chain”. Immunity (2016) 45:225–6. doi: 10.1016/j.immuni.2016.07.006 27533005

[26] Kao D Danzer H Collin M Gross A Eichler J Stambuk J. A monosaccharide residue is sufficient to maintain mouse and human IgG subclass activity and directs IgG effector functions to cellular fc receptors. Cell Rep (2015) 13:2376–85. doi: 10.1016/j.celrep.2015.11.027 26670049

[27] Schwab I Lux A Nimmerjahn F. Pathways responsible for human autoantibody and therapeutic intravenous IgG activity in humanized mice. Cell Rep (2015) 13:610–20. doi: 10.1016/j.celrep.2015.09.013 26456831

[28] Chester C Sanmamed MF Wang J Melero I. Immunotherapy targeting 4-1BB: mechanistic rationale, clinical results, and future strategies. Blood (2018) 131:49–57. doi: 10.1182/blood-2017-06-741041 29118009

[29] Hurtado JC Kim SH Pollok KE Lee ZH Kwon BS. Potential role of 4-1BB in T cell activation. comparison with the costimulatory molecule CD28. J Immunol (1995) 155:3360–7. 7561030

[30] Hurtado JC Kim YJ Kwon BS. Signals through 4-1BB are costimulatory to previously activated splenic T cells and inhibit activation-induced cell death. J Immunol (1997) 158:2600–9. 9058792

[31] Kwon BS Weissman SM. cDNA sequences of two inducible T-cell genes. Proc Natl Acad Sci U.S.A. (1989) 86:1963–7. doi: 10.1073/pnas.86.6.1963 PMC286825 2784565

[32] Lux A Nimmerjahn F. Of mice and men: the need for humanized mouse models to study human IgG activity *in vivo* . J Clin Immunol (2013) 33 Suppl 1:S4–8. doi: 10.1007/s10875-012-9782-0 22948744

[33] Chen H Maul-Pavicic A Holzer M Huber M Salzer U Chevalier N. Detection and functional resolution of soluble immune complexes by an FcgammaR reporter cell panel. EMBO Mol Med (2022) 14:e14182. doi: 10.15252/emmm.202114182 34842342 PMC8749491

[34] Heidelberger M Kendall FE. A quantitative study of the precipitin reaction between type iii pneumococcus polysaccharide and purified homologous antibody. J Exp Med (1929) 50:809–23. doi: 10.1084/jem.50.6.809 PMC2131658 19869667

[35] Baerenwaldt A Lux A Danzer H Spriewald BM Ullrich E Heidkamp G. Fcgamma receptor IIB (FcgammaRIIB) maintains humoral tolerance in the human immune system *in vivo* . Proc Natl Acad Sci U.S.A. (2011) 108:18772–7. doi: 10.1073/pnas.1111810108 PMC3219118 22065769

[36] Albert H Collin M Dudziak D Ravetch JV Nimmerjahn F. *In vivo* enzymatic modulation of IgG glycosylation inhibits autoimmune disease in an IgG subclass-dependent manner. Proc Natl Acad Sci U.S.A. (2008) 105:15005–9. doi: 10.1073/pnas.0808248105 PMC2567483 18815375

