# Supplementary material for: Modulation of urelumab glycosylation separates immune stimulatory activity from organ toxicity
Source: Front Immunol. 2022 Sep 29;13:970290. doi: 10.3389/fimmu.2022.970290 (PMC9558126; doi:10.3389/fimmu.2022.970290)
Supplement: Supplementary Figure 1 — Identification of human immune cell subsets in humanized mice via flow cytometry. Shown is an exemplary gating strategy separating mouse immune cells (mCD45+) from human immune cells (hCD45+) in splenic single cell preparations of humanized mice three months after HSC transplantation. Human immune cell subsets (hCD45+) were identified by expression of human immune cell lineage specific markers, such as CD3, CD4, and CD8 to detect human CD8+ and CD4+ T cell subsets or CD56 or CD33 to identify natural killer (NK) cells or monocytes, respectively. CD16 in combination with CD56, as well as CD16/CD14 in combination with CD33 was used to identify further subsets of NK cells or monocytes. [file Presentation_1.pdf]

Supplementary Figures

Title: Modulation of urelumab glycosylation separates immune stimulatory activity from organ toxicity

Authors: Carmen Reitinger, Andrea Ipsen-Escobedo, Chiara Hornung, Lukas Heger, Diana Dudziak, Anja Lux, and Falk Nimmerjahn

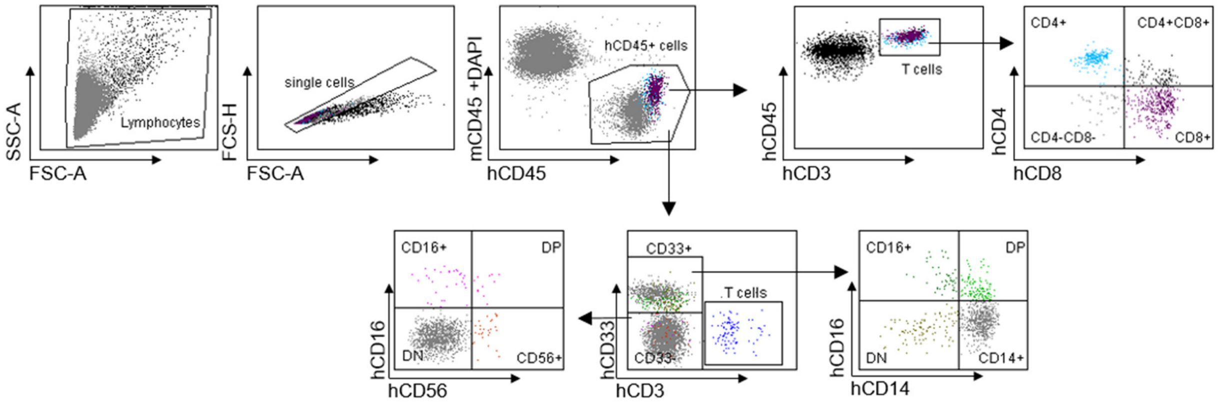

Supplementary Figure S1: Identification of human immune cell subsets in humanized mice via flow cytometry. Shown is an exemplary gating strategy separating mouse immune cells (mCD45+) from human immune cells (hCD45+) in splenic single cell preparations of humanized mice three months after HSC transplantation. Human immune cell subsets (hCD45+) were identified by expression of human immune cell lineage specific markers, such as CD3, CD4, and CD8 to detect human CD8+ and CD4+ T cell subsets or CD56 or CD33 to identify natural killer (NK) cells or monocytes, respectively. CD16 in combination with CD56, as well as CD16/CD14 in combination with CD33 was used to identify further subsets of NK cells or monocytes.

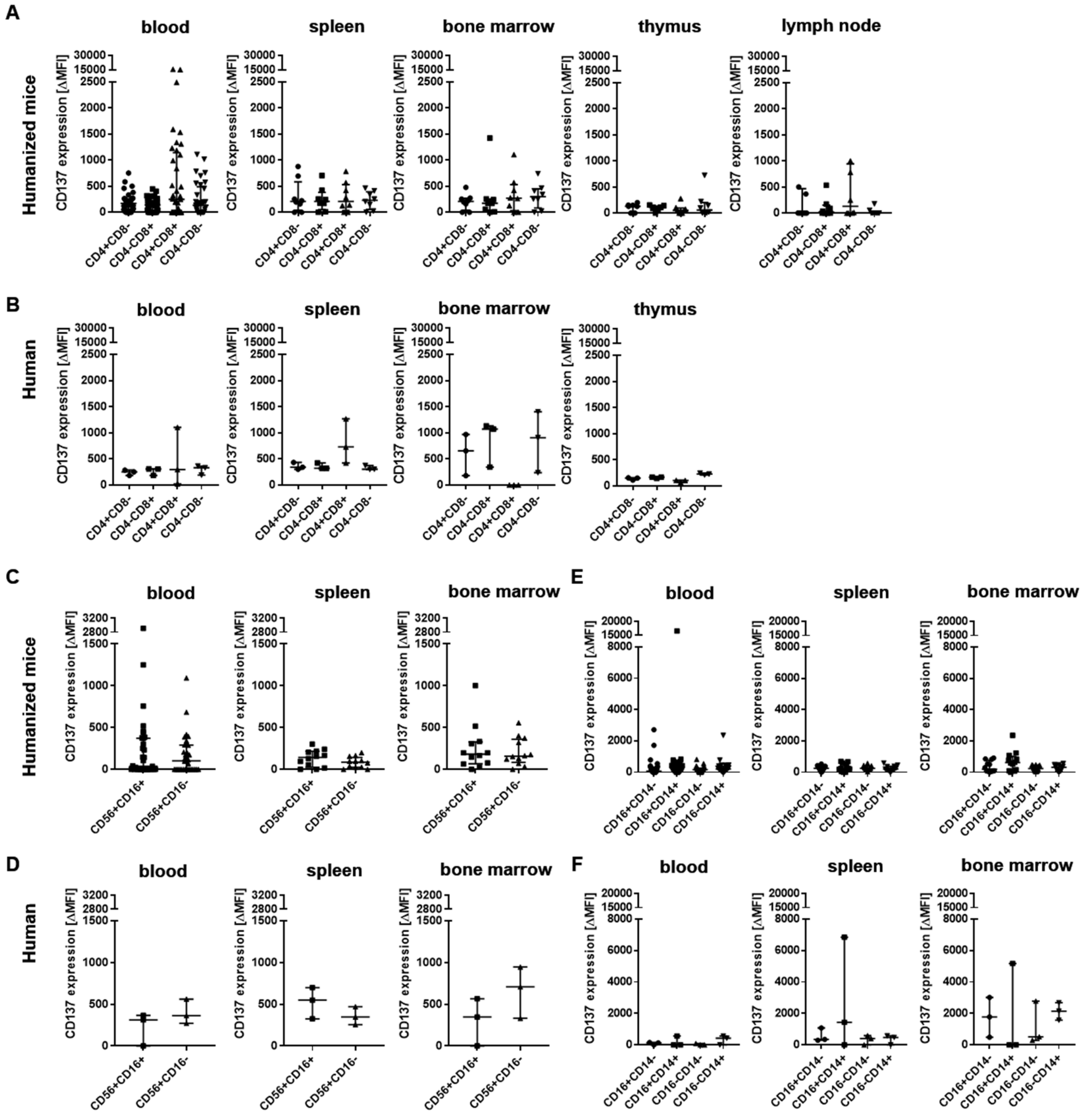

Supplementary Figure S2: Comparison of CD137 expression on human T cells, NK cells and monocytes in humanized mice and humans. (**A**) Shown is CD137 expression on $CD3^{+}$ T cell subsets in humanized mice in blood (n=37), spleen, bone marrow, thymus and lymph node (n=8) samples. (**B**) Shown is CD137 expression on the indicated human $CD3^{+}$ T cell subsets in human peripheral blood, spleen, bone marrow as well as on thymic T cells (n=3). (**C, D**) Shown is CD137 expression on NK cell subsets in the respective organs in humanized mice (blood (n=33), spleen and bone marrow (n= 12)) (**C**) and humans (n=3) (**D**). (**E, F**)

Depicted is CD137 expression on $CD33^+$ monocyte subsets located within the indicated organs in humanized mice (blood: n=33, spleen and bone marrow: n= 12) (**E**), as well as in the respective human organs (**F**) (n=3). Results are expressed as median of ΔMFI with interquartile range.

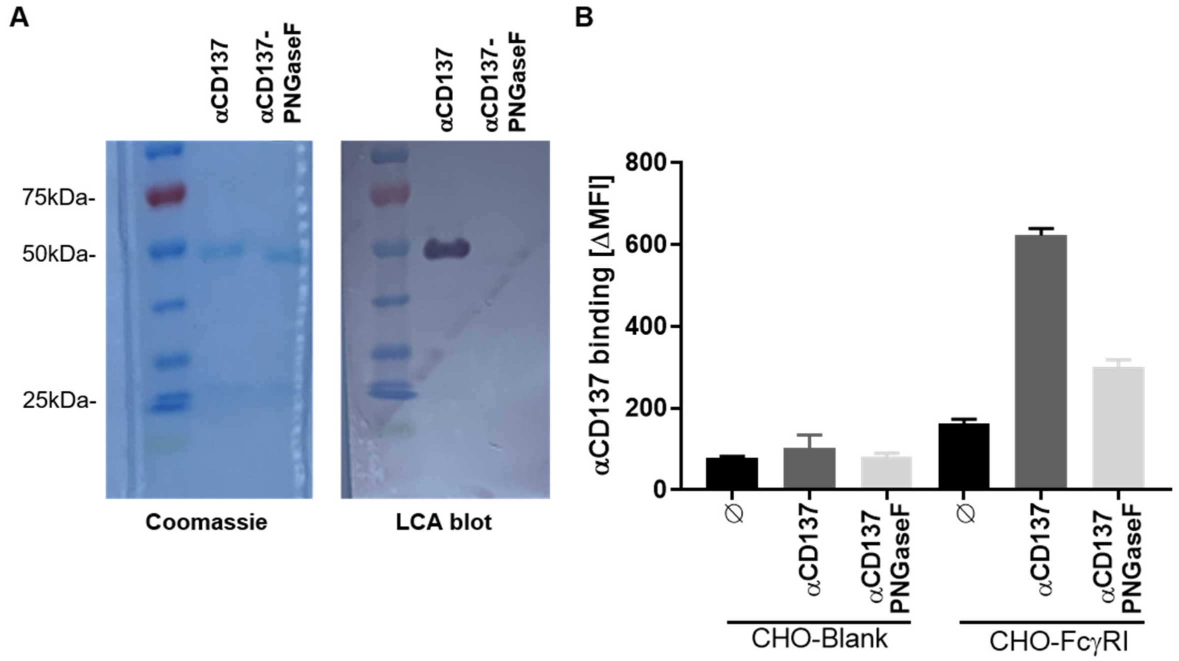

Supplementary Figure S3: Effect of urelumab deglycosylation on binding to human FcγRI. (**A**) Shown is a Coomassie stained PAA gel (left panel) and a western blot analysis (right panel) using lens culinaris agglutinin (LCA) to detect the effect of deglycosylation on urelumab size or the urelumab core sugar structure, respectively. (**B**) Depicted is the binding of untreated urelumab (αCD137) or of PNGaseF treated urelumab (αCD137 PNGaseF) to CHO cells (CHO-Blank) or CHO cells expressing human FcγRI (CHO-FcγRI) as the delta mean fluorescence intensity (ΔMFI) as determined by flow cytometry. Bound urelumab was detected with fluorescently labelled protein L. As a further control, CHO cells as well as CHO-FcγRI expressing cells were stained with protein L in the absence of urelumab variants (∅). Shown is the mean +/- SEM of two independent experiments.

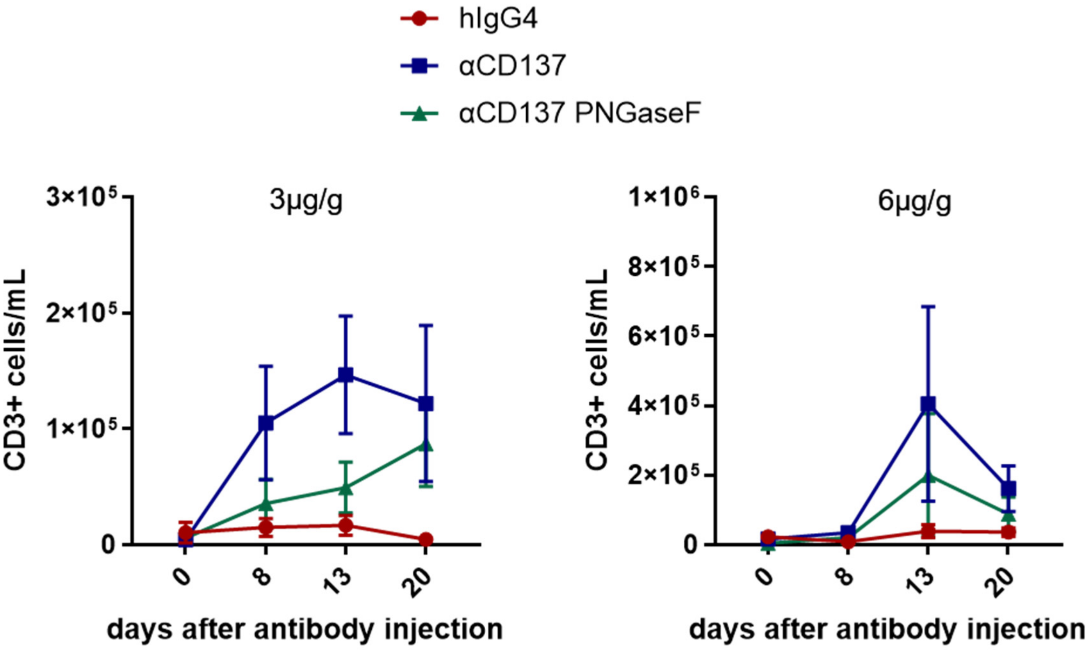

Supplementary Figure S4: Effect of urelumab variants on absolute human T cell numbers in the peripheral blood. Shown are the absolute numbers of CD3+ human T cells in the peripheral blood of humanized mice upon treatment with 3μg/g (hIgG4: n= 5; αCD137: n=6; αCD137 PNGaseF: n=4) or 6μg/g (hIgG4: n= 7-13; αCD137: n=10-11; αCD137 PNGaseF: n=6) of a human IgG4 isotype control antibody, urelumab (αCD137) or a PNGaseF treated urelumab variant (αCD137 PNGaseF). For each time point the mean+/-SEM is shown.

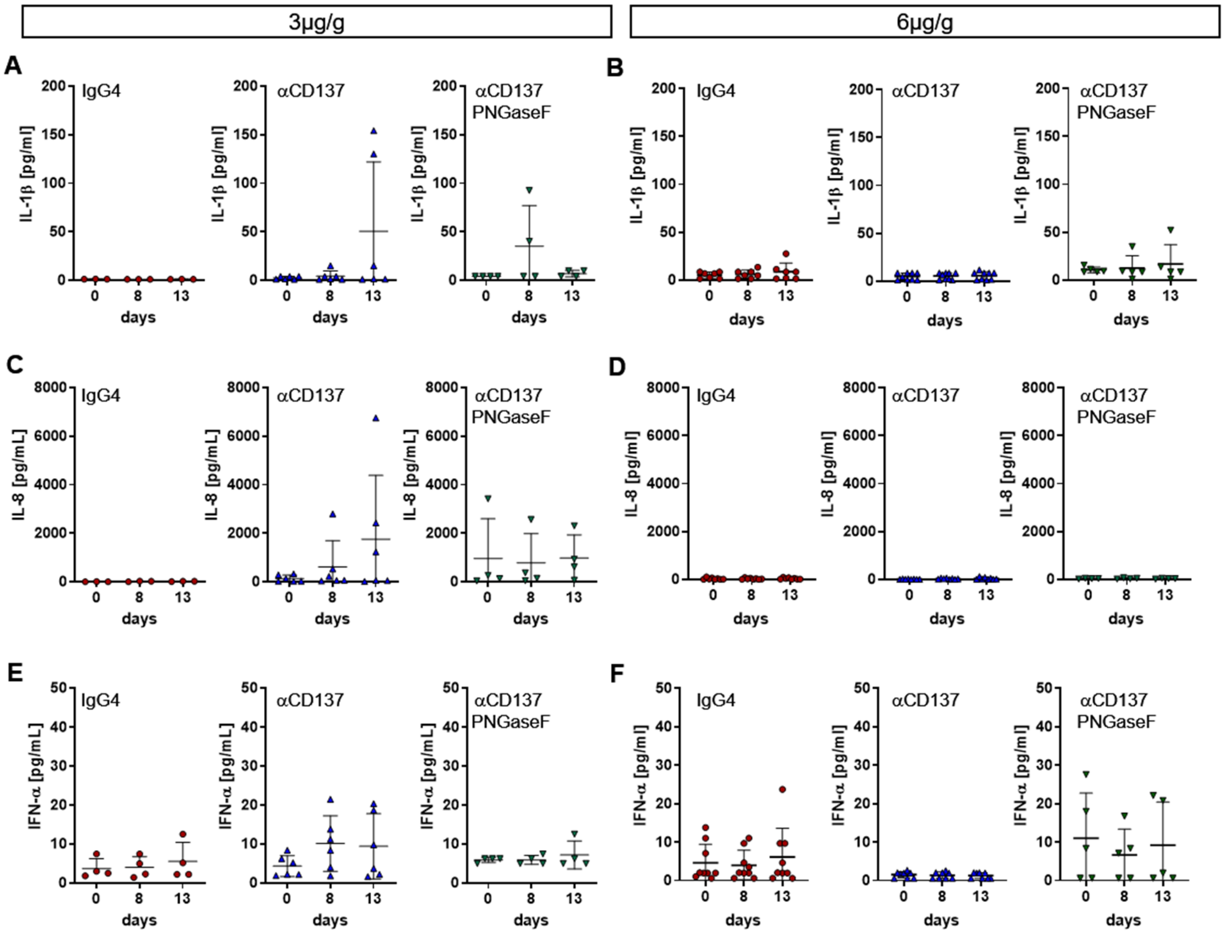

Supplementary Figure S5: Effect of urelumab variant injection on serum cytokine levels. Serum cytokine concentrations were determined before and at the indicated time-points after treating humanized mice with 3µg/g (**A, C, E**) or 6µg/g (**B, D, F**) of hIgG4, αCD137 or with the deglycosylated αCD137 variant (αCD137 PNGaseF). Depicted are serum concentrations of (**A**) IL-1β (hIgG4: n=3, aCD137: n=6, αCD137 PNGaseF: n=4), (**C**) IL-8 (hIgG4: n=3, αCD137: n=6, αCD137 PNGaseF: n=4), and (**E**) IFN-α (hIgG4: n=4, αCD137: n=6, aCD137 PNGaseF: n=4) in mice treated with 3µg/g of the indicated antibody variants. Concentration of (**B**) IL-1β (hIgG4: n=7, aCD137: n=9, aCD137 PNGaseF: n=5), (**D**) IL-8 (hIgG4: n=8, αCD137: n=8, αCD137 PNGaseF: n=4), and (**F**) IFN-α (hIgG4: n=9, αCD137: n=8, αCD137 PNGaseF: n=5), in mice treated with 6µg/g of antibody variants. Results are expressed as median of ∆MFI with interquartile range. A ROUT outlier test (Q=1%) and Shapiro-Wilk normality test were performed followed by either Friedman test with Dunn‘s multiple comparison test or RM-One-Way ANOVA with Tukey‘s multiple comparison test was performed.
